# Subchronic Oral Toxicity of Sodium p-Hydroxybenzoate in Sprague-Dawley Rats

**DOI:** 10.3389/fphar.2022.843368

**Published:** 2022-03-09

**Authors:** Xiaoli Fan, Hengzhi Song, Xiaotian Xu, Xi Lu, Yuhui Wang, Xiaoqun Duan

**Affiliations:** ^1^ Department of Pharmacy, Xiangya Hospital, Central South University, Changsha, China; ^2^ College of Pharmacy, Guilin Medical University, Guilin, China

**Keywords:** sodium p-hydroxybenzoate, toxicity, NOAEL, safety assessment, p-hydroxybenzoic acid

## Abstract

p-Hydroxybenzoic acid (p-HBA), which exists extensively in plants, is well known for its anti-inflammatory effects, but various adverse side effects have also been reported. Previous research has found that acid translated to its sodium salt improves the safety profile of compounds. Therefore, we hypothesized that p-HBA translated to sodium p-hydroxybenzoate would improve its safety profile. In the present study, we evaluated the toxicity of sodium p-hydroxybenzoate after 90 days of repeated oral toxicity experiments according to OECD guidelines in male and female Sprague–Dawley rats. Sodium p-hydroxybenzoate was administered orally to SD rats at doses of 0, 125, 250, and 500 mg/kg body weight (BW)/day for 90 days. All animals survived to the end of the study, and no sodium p-hydroxybenzoate treatment-associated mortality or clinical changes were observed during the study period. Sodium p-hydroxybenzoate did not promote any clinical signs of toxicologically relevant effects, including changes in body weight, food intake and urinalysis parameters, in male or female SD rats. Dose-related alterations in hematological parameters, organ weights and histopathological findings in hepatic tissue were examined in animals of both sexes in the 500 mg/kg BW/day group. Based on the study, the no-observed-adverse-effect level (NOAEL) for sodium p-hydroxybenzoate was determined to be 250 mg/kg BW/day in both male and female rats.

## Introduction

p-Hydroxybenzoic acid (p-HBA), a monohydroxy phenolic derivative of phenolic acid containing a C6-C1 carbon skeleton, exists extensively in plants (Kim et al., 2020). Various studies have confirmed that p-HBA has therapeutic advantages such as antibacterial (Qin et al., 2020; Liu et al., 2021), anticancer (Spatafora and Tringali, 2012), antioxidative (Winter et al., 2017) and anti-inflammatory activities (Rakesh et al., 2018; Sabater et al., 2019). Previously, our research group found p-HBA was more effective than mesalazine in DSS-induced ulcerative colitis (UC) of C57BL/6 mice. However, the safety profile of p-HBA limits its use for developing new drugs; for example, the oral 50% lethal dose (LD50) of p-HBA in rats is approximately 2,000 mg/kg, and adverse effects in response to p-HBA have been reported, including infertility in males and breast cancer in females (Asif et al., 2021). Therefore, maintaining the pharmaceutical effect while improving the safety of p-HBA would facilitate the development of clinical drugs in the future.

Previous research has revealed that acid translation to its sodium salt would represent a good approach for improving the safety profile and stability of compounds, such as diclofenac. Diclofenac is a nonsteroidal anti-inflammatory drug (Coxib et al.) of the phenylacetic acid class and is associated with serious gastrointestinal and renal adverse effects (Kuo et al., 2010; CNT et al., 2013). With the goal of improving the safety profile of diclofenac, delayed- and extended-release forms of diclofenac sodium were developed (Altman et al., 2015). Therefore, we speculated that the safety profile would be improved and that the pharmaceutical effect would be maintained if p-HBA was translated into sodium p-hydroxybenzoate.

Sodium benzoate is typically used as an antimicrobial additive for food and in the pharmaceutical industry (Yadav et al., 2016). However, there are limited references regarding the toxicological and safety information of sodium p-hydroxybenzoate. In our previous acute oral toxicity experiments, the LD50 of sodium p-hydroxybenzoate was greater than 5,000 mg/kg. Therefore, in the present study, we investigated the toxicity of sodium p-hydroxybenzoate using a 90-days subchronic oral toxicity test in Sprague–Dawley (SD) rats to determine toxicological profiles and establish the no-observed-adverse-effect level (NOAEL). This study represents the first safety profiling of sodium p-hydroxybenzoate in accordance with the Economic Cooperation and Development (OECD) guideline 408 (OECD, 2018). Our findings provide an imperative reference for evaluating the safety of sodium p-hydroxybenzoate.

## Materials and Method

### Reagents

**FIGURE 1:**
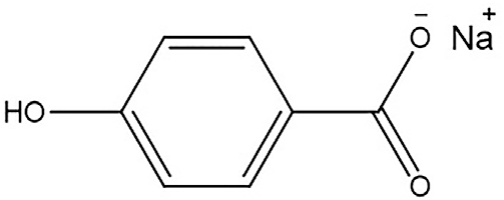
Chemical structure of sodium p-hydroxybenzoate.

Sodium p-hydroxybenzoate (LOT NO.: Y10S6K3245, purity: 98.5%) (Figure 1) was purchased from Shanghai Yuanye Bio-Technology Co., Ltd. (Shanghai, China). In this experiment, sodium p-hydroxybenzoate was suspended in distilled water by vortexing and sonication, and dose formulations were prepared on the day of administration.

### Experimental Animals and Their Maintenance

Eighty male and female SD rats (40 each; 5 weeks old) were obtained from Hunan SJA Laboratory Animal Co. Ltd. under license number SCXK (YUE) 2008–2002 and were used for the 90-days subchronic toxicity study. All procedures in this experiment were strictly reviewed and approved by the Animal Ethics Committee of Guilin Medical University (animal protocol approval number, GLMC201703011). The animals were acclimated for at least 1 week to the laboratory environment before the study, and then they were housed individually in stainless steel wire mesh cages.

At the beginning of the study, all animals were healthy, the body weights were within ±20% of the mean body weight for each sex, and the body weight range at 6 weeks of age was 184–222 g for males (mean 206.5 g) and 160–209 g for females (mean 182.95 g). The animals were housed in an animal room under controlled environmental conditions: 20–22 °C with a relative humidity of 55–65%, air ventilation 18 times per hour, and a 12 h light/dark cycle with standard diet and water. The cages and padding were changed twice a week. Before the experiments, healthy SD rats were randomly divided into four groups with 10 females and 10 males each and measured before starting the administration.

### Study Design and Dose Level

This study was performed in accordance with the OECD Test Guidelines (Test NO. 408: repeated dose 90-days oral toxicity study in rodents) with slight modifications. This study was performed at the Animal Center of Guilin Medical University, and the dose volume administered during the subchronic oral toxicity study was determined by the acute oral toxicity study. Sodium p-hydroxybenzoate was dissolved in distilled ultrapure water and then administered to animals via oral gavage once daily for a period of 90 consecutive days at concentrations of 0, 125, 250, and 500 mg/kg BW/day in a volume of 10 ml/kg, and the vehicle served as a control.

During the study period, all animals were examined for signs of morbidity and mortality twice a day. General clinical observations were made and recorded at the same time once a day, and detailed clinical observations were made of all animals once a week. These observations were made outside the home cage and were carefully recorded. Observations included behavioral patterns, physical appearance, and other toxicity-related symptoms. The home cage observations included posture, convulsions, and abnormal behavior.

### Body Weight and Food Intake

Body weights were recorded for all animals on the first day of the experiment and subsequently once a week thereafter, and the measurement of feed consumption was monitored weekly throughout the entire study.

### Hematology and Clinical Biochemistry

At the end of the study, all rats were fasted overnight before collecting blood samples for hematology and clinical biochemistry. Blood samples were obtained from the hearts of animals by intracardiac withdrawal. The hematology parameters were analyzed using an automatic hematology analyzer (XN-9000, Sysmex Corp. Japan), including hemoglobin concentration (HGB), red blood cell count (RBC), mean corpuscular volume (MCV), mean corpuscular hemoglobin (MCH), mean corpuscular hemoglobin concentration (MCHC), hematocrit value (HCT), white blood cell count (WBC), red blood cell distribution width (RDW), platelet count (PLT), white blood cell count (WBC), and differential leukocyte count including neutrophils (NEUT), eosinophils (EOS), basophils (BASO), monocytes (MONO) and lymphocytes (LYM).

Blood samples for coagulation tests were used to determine the prothrombin time (PT) and activated partial thromboplastin time (APTT) using a blood coagulation analyzer (CA-1500, Sysmex Corp. Japan).

The following serum biochemistry parameters were evaluated using an automatic chemistry analyzer (Cobas 8,000, Roche Diagnostics, IN, United States): aspartate aminotransferase (AST), alanine aminotransferase (Altman et al.), alkaline phosphatase (ALP), total bile acid (TBA), total cholesterol (TCHO), high-density lipoprotein (HDL), low-density lipoprotein (LDL), triglyceride (TG), phospholipid (PL), total bilirubin (T-BIL), glucose (GLU), blood urea nitrogen (BUN), urea, creatinine (CRNN), total protein (TP), albumin (ALB), globulin, A/G ratio (A/G), sodium (Na), potassium (K) and chloride (Cl).

Serum thyroid-stimulating hormone levels of triiodothyronine (T3), thyroxine (T4) and thyroid-stimulating hormone (TSH) were assessed using an automatic chemiluminescence immunoassay analyzer (COBAS 8000 e801, Roche Diagnostics, IN, United States).

### Urinalysis

At the end of the study, a metabolic cage used for urine collection was used for urinalysis. Urine pH, density, protein, ketones, glucose, occult blood, urobilinogen, bilirubin, urinary sediments and cast were analyzed using a Toshiba 200FR NEO chemistry analyzer (Toshiba Co., Tokyo, Japan).

### Organ Weight and Pathology Examination

After collecting the blood samples, all rats were subjected to gross necropsy. Then, the primary organs were excised and weighed, such as the brain, heart, spleen, liver, lungs, kidneys, adrenal glands, thymus, testis (male), epididymis (male), ovaries (female) and uterus (female). The relative organ weights of each rat were then calculated (organ weight as a % of body weight).

In addition to these organs, trachea, thyroid, parathyroid gland, aorta, esophagus, stomach, small and large intestines, pancreas, urinary bladder, prostate, cervical and mesenteric lymph nodes, sternum, sciatic nerve and mammary glands were fixed in 10% neutral-buffered formalin. According to the recommendations of the OECD Guideline 407 (OECD, 2008), all preserved organs and tissues in the control and highest dose groups (500 mg/kg group) were subjected to full histopathology. Tissues were routinely made into paraffin-embedded sections and stained with hematoxylin and eosin (HE) for histopathological evaluation. If treatment-related changes appeared at the highest dose, the relevant tissues from the lower dose groups were also examined.

### Statistical Analysis

All experimental data and statistical analyses were performed using the Statistical Package for the Social Sciences (SPSS) software version 22.0 (IBM, Armonk, NY, United States). Levene’s test was used to test the assumption of homogeneity of variance. If the assumption of homogeneity of variance was met, the post hoc Bonferroni test was used to determine which groups that were significantly different from the control group. When statistically significant differences were observed, the Dunnett T3 test was employed for comparisons between the control and treatment groups. All data are presented as the mean ± standard deviation in each group, and *p*-values < 0.05 were considered statistically significant.

## Result

### Mortality and Clinical Signs

All animals survived, and no abnormal clinical signs associated with sodium p-hydroxybenzoate treatment were observed among any of the groups in either females or males. Specifically, compared to the control group, SD rats exhibited no significant abnormalities on ophthalmology examination in either sex. Furthermore, no abnormal clinical signs or changes were found in any treated SD rats in the clinical observations made outside the home cage.

### Body Weight and Feed Consumption

**FIGURE 2:**
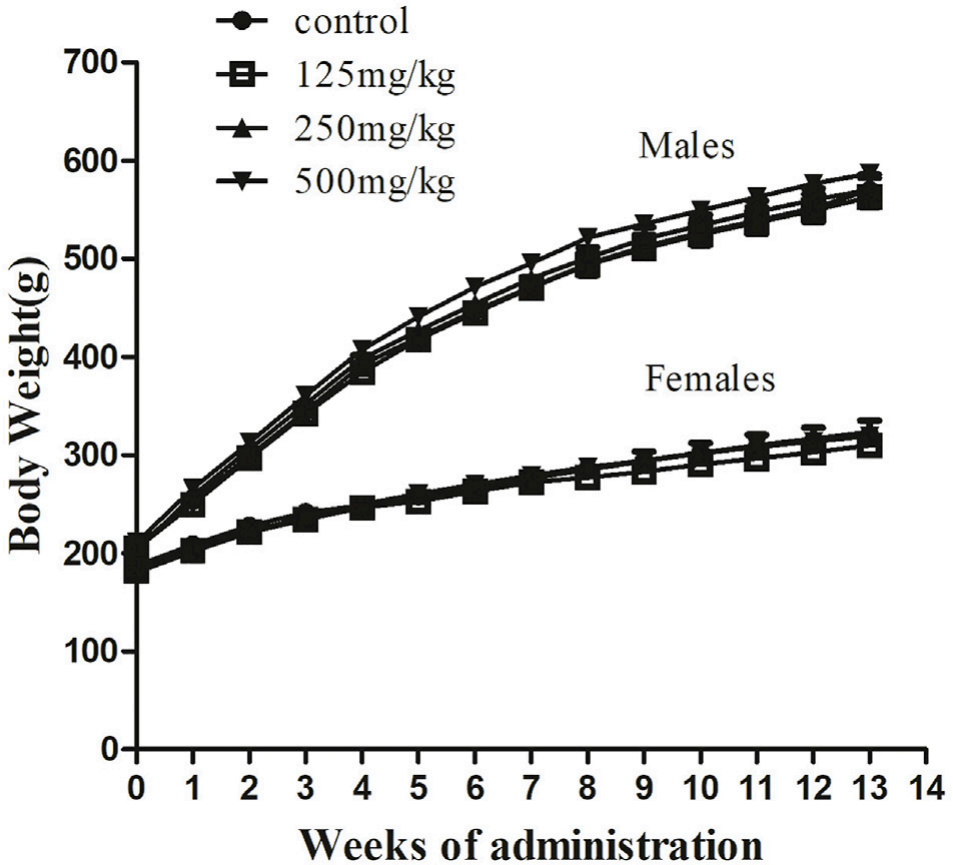
Body weight evolution in male and female SD rats treated with sodium p-hydroxybenzoate in the 90-days subchronic toxicity study. Data are shown as the means ± SD. One-way ANOVA and Bonferroni tests were used to compare results between groups at each time point. *p* < 0.05 was considered significant.

**FIGURE 3:**
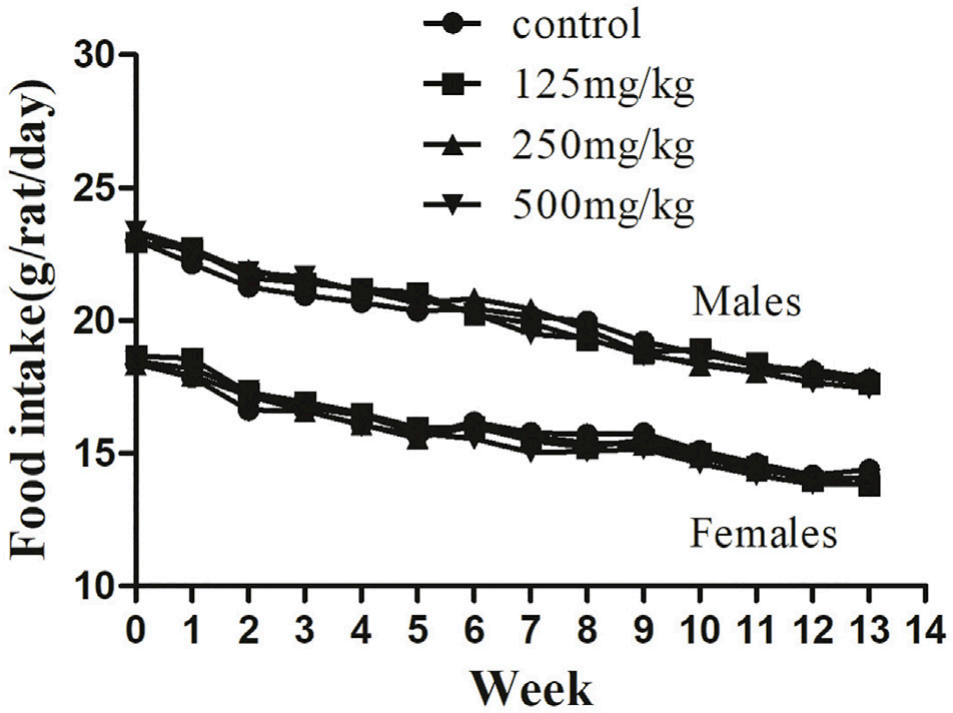
Feed consumption in male and female SD rats treated with sodium p-hydroxybenzoate in the 90-days subchronic toxicity study. Data are shown as the means ± SD. One-way ANOVA and Bonferroni tests were used to compare results between groups at each time point. *p* < 0.05 was considered significant.

To further study the influence of sodium p-hydroxybenzoate on the rat model, we recorded changes in body weight (Figure 2) and food consumption (Figure 3) in the presence and absence of sodium p-hydroxybenzoate. Regardless of sex, all rats gained weight, and body weight increased in a time-dependent manner throughout the study. No significant differences in body weight were noted between the control and treated groups of male and female rats throughout the 90-days experimental period.

Similarly, the food consumption presented in Figure 3 shows no difference between the control and sodium p-hydroxybenzoate-treated groups. All experimental subjects exhibited a similar and slightly decreasing trend in feed consumption in both male and female rats in all groups during the study period.

### Hematological Parameters

Hematology-related markers are important indicators of pathophysiological status (Li et al., 2021). Therefore, to further determine the pathophysiological status of the animals, we next examined the hematological parameters of the rats. The influence of sodium p-hydroxybenzoate on the hematology parameters of male and female rats is shown in Table 1; Table 2, respectively. First, compared to the control group, white blood cells (WBCs) in the male group at a dose of 500 mg/kg increased significantly, but this trend was not detected in female rats of any dose group. Furthermore, a significant (*p* < 0.05) decrease in the coagulation parameter prothrombin time (PT) was observed in the 500 mg/kg BW/day dose group compared to the control group. In females, no significant difference was observed in hematological parameters of any of the dose groups. In comparison, the 500 mg/kg BW/day dose significantly increased WBCs, which are regarded as an effective marker of inflammation (Karino et al., 2015), indicating that the 500 mg/kg dose may cause hematological toxicity in male rats. PT is a significant coagulation biomarker due to its direct correlation to the physiological clotting time, and prolonged PT represents impairment of coagulation function (Saha et al., 2021). In this study, since the PT did not change in the 500 mg/kg BW/day dose group in females, this was considered to be due to individual differences.

**TABLE 1:** Hematological parameters of male SD rats treated with sodium p-hydroxybenzoate for 90 days.

Parameter	sodium p-hydroxybenzoate (mg/kg)			
	0	125	250	500
NO. of animals examined	10	10	10	10
Males				
RBC (10^6/μL)	9.47 ± 0.317	9.49 ± 0.47	8.99 ± 0.35	9.06 ± 0.83
HGB (g/L)	173.0 ± 14.93	169.8 ± 13.2	159.5 ± 9.87	164.4 ± 18.52
HCT (%)	52.76 ± 6.14	52.68 ± 2.68	49.48 ± 2.22	50.78 ± 4.21
MCV (fL)	55.6 ± 5.23	55.52 ± 0.60	55.02 ± 1.04	56.08 ± 1.29
MCH (pg)	18.24 ± 1.10	17.91 ± 1.04	17.72 ± 0.84	18.13 ± 1.28
MCHC (g/L)	329.1 ± 16.45	322.5 ± 16.15	322.1 ± 12.55	323.0 ± 17.60
RDW (%)	13.2 ± 0.59	13.69 ± 0.68	13.83 ± 0.57	13.26 ± 0.56
PLT (10^4/μL)	116.24 ± 8.98	119.49 ± 22.40	110.18 ± 11.78	107.68 ± 12.45
WBC (10^3/μL)	8.12 ± 0.97	9.36 ± 1.59	9.22 ± 0.66	9.68 ± 1.32*
Neu (10^3/μL)	1.84 ± 0.45	1.79 ± 0.23	1.812 ± 0.28	1.871 ± 0.64
Lym (10^3/μL)	6.93 ± 1.76	8.37 ± 2.14	7.576 ± 1.13	7.629 ± 0.69
Mon (10^3/μL)	0.275 ± 0.104	0.309 ± 0.109	0.325 ± 0.087	0.379 ± 0.156
EOS (10^3/μL)	0.132 ± 0.053	0.135 ± 0.058	0.128 ± 0.043	0.134 ± 0.055
Bas (10^3/μL)	0.026 ± 0.019	0.023 ± 0.016	0.024 ± 0.019	0.018 ± 0.013
PT (sec)	14.90 ± 0.67	14.26 ± 0.66	14.68 ± 0.95	13.76 ± 0.84*
APTT (sec)	15.86 ± 1.21	16.11 ± 0.98	16.44 ± 0.72	16.09 ± 1.30

RBC, red blood cell count; HGB, hemoglobin; HCT, hematocrit; MCV, mean corpuscular volume; MCH, mean corpuscular hemoglobin; MCHC, mean corpuscular hemoglobin concentration; RDW%, red blood cell distribution width; PLT, platelet count; WBC, white blood cell count; NEU, neutrophil count; LYM, lymphocyte count; MON, monocyte count; EOS, eosinophil count; BAS, basophil count; PT, prothrombin time; APTT, activated partial thromboplastin time.

Data are expressed as means ± SD. *p < 0.05, (significantly different from the control group).

One-way ANOVA test and Bonferroni test.

**TABLE 2:** Hematological parameters of female SD rats treated with sodium p-hydroxybenzoate for 90 days.

Parameter	sodium p-hydroxybenzoate (mg/kg)			
	0	125	250	500
NO. of animals examined	10	10	10	10
Females				
RBC (10^6/μL)	8.10 ± 0.28	7.97 ± 0.75	7.99 ± 0.40	7.76 ± 0.99
HGB (g/L)	158.4 ± 9.62	157.2 ± 13.30	153.9 ± 13.05	154.4 ± 19.50
HCT (%)	47.41 ± 1.78	47.88 ± 3.95	46.24 ± 2.90	46.31 ± 5.96
MCV (fL)	58.56 ± 0.96	60.19 ± 2.58	57.84 ± 1.38	59.74 ± 1.87
MCH (pg)	19.55 ± 0.98	19.76 ± 0.36	19.22 ± 0.93	19.94 ± 0.72
MCHC (g/L)	333.9 ± 11.73	328.6 ± 9.85	332.2 ± 10.84	333.7 ± 7.57
RDW (%)	13.64 ± 0.33	13.79 ± 1.20	13.34 ± 0.51	13.7 ± 1.26
PLT (10^4/μL)	101.8 ± 5.94	102.1 ± 8.86	107.0 ± 3.89	101.9 ± 7.21
WBC (10^3/μL)	8.99 ± 0.86	8.82 ± 1.78	8.73 ± 0.96	8.63 ± 1.94
Neu (10^3/μL)	0.86 ± 0.26	0.97 ± 0.38	1.04 ± 0.35	0.83 ± 0.28
Lym (10^3/μL)	3.75 ± 1.06	3.64 ± 0.51	4.12 ± 1.63	4.04 ± 1.15
Mon (10^3/μL)	0.202 ± 0.156	0.169 ± 0.060	0.186 ± 0.059	0.198 ± 0.097
EOS (10^3/μL)	0.082 ± 0.019	0.074 ± 0.016	0.09 ± 0.022	0.089 ± 0.026
Bas (10^3/μL)	0.017 ± 0.013	0.014 ± 0.010	0.021 ± 0.017	0.018 ± 0.015
PT (sec)	14.65 ± 1.10	14.02 ± 1.16	15.26 ± 1.15	14.34 ± 1.03
APTT (sec)	15.48 ± 0.87	15.06 ± 1.15	14.95 ± 1.14	15.95 ± 0.83

RBC, red blood cell count; HGB, hemoglobin; HCT, hematocrit; MCV, mean corpuscular volume; MCH, mean corpuscular hemoglobin; MCHC, mean corpuscular hemoglobin concentration; RDW%, red blood cell distribution width; PLT, platelet count; WBC, white blood cell count; NEU, neutrophil count; LYM, lymphocyte count; MON, monocyte count; EOS, eosinophil count; BAS, basophil count; PT, prothrombin time; APTT, activated partial thromboplastin time.

Data are expressed as means ± SD. *p < 0.05, (significantly different from the control group).

One-way ANOVA test and Bonferroni test.

### Clinical Biochemistry Parameters

The serum biochemical parameters of male and female rats are shown in Table 3 and Table 4, respectively. Compared to the control group, a reduction in urea (*p* < 0.05) was only found in the 250 mg/kg BW/day male group, which is considered incidental and not dose-related because no differences were noted in the other groups. In addition, male subjects (the 500 mg/kg BW/day group) exhibited a significant (*p* < 0.05) decrease in total cholesterol compared to the control group, while no differences were observed in females. Despite attaining statistical significance, the cholesterol value was within normal ranges for rats. In females, total bilirubin was significantly decreased in the 500 mg/kg BW/day group compared to the control group, while no differences were noted in males. Increased bilirubin levels are associated with primary biliary cirrhosis and hepatic cholestasis (Thapa and Walia, 2007), so a decrease in total bilirubin might have a protective effect. Therefore, these changes were considered to be nonadverse and not of toxicological concern because these alterations did not occur in both sexes and were not dose-related.

**TABLE 3:** Biochemical parameters of male SD rats treated with sodium p-hydroxybenzoate for 90 days.

Parameter	sodium p-hydroxybenzoate (mg/kg)			
	0	125	250	500
NO. of animals examined	10	10	10	10
Males				
ALT (U/L)	29.3 ± 3.59	30.7 ± 2.21	31.0 ± 6.62	27.3 ± 1.77
AST (U/L)	96.9 ± 2.85	95.3 ± 5.83	96.0 ± 10.47	93.1 ± 5.97
Glucose (mmol/L)	6.51 ± 0.25	7.01 ± 0.89	6.57 ± 1.31	6.77 ± 0.76
Urea (mmol/L)	7.34 ± 1.51	7.07 ± 0.79	5.97 ± 0.79*	7.02 ± 0.89
Creatinine (μmol/L)	43.3 ± 7.57	40.7 ± 3.34	40.2 ± 2.10	42.9 ± 3.07
Total cholesterol (mmol/L)	1.74 ± 0.17	1.71 ± 0.20	1.65 ± 0.35	1.43 ± 0.21*
Triglyceride (mmol/L)	1.25 ± 0.20	1.10 ± 0.13	1.32 ± 0.10	1.22 ± 0.10
Total Bilirubin (μmol/L)	1.84 ± 0.12	1.81 ± 0.09	1.67 ± 0.34	1.68 ± 0.35
Total Protein (g/L)	63.6 ± 2.80	62.1 ± 1.66	61.7 ± 1.64	61.4 ± 2.88
Albumin (g/L)	41.7 ± 1.83	42.4 ± 1.43	41.1 ± 3.21	41.3 ± 1.57
Globulins (g/L)	21.9 ± 2.85	19.7 ± 2.36	20.6 ± 2.67	20.1 ± 2.60
A/G (%)	1.94 ± 0.28	2.19 ± 0.32	2.04 ± 0.36	2.12 ± 0.29
Na (mmol/L)	143.1 ± 2.97	143.24 ± 3.99	143.1 ± 1.53	144.3 ± 2.04
K (mmol/L)	4.32 ± 0.60	4.52 ± 0.54	4.64 ± 0.54	4.57 ± 0.62
Cl (mmol/L)	101.3 ± 6.55	104.1 ± 6.01	102.7 ± 7.01	103.3 ± 5.66

AST, aspartate aminotransferase; ALT, alanine aminotransferase; A/G, albumin/globulin ratio; K, potassium; Na, sodium; Cl, chloride.

Data are expressed as means ± SD. *p < 0.05, (significantly different from the control group).

One-way ANOVA test and Bonferroni test.

**TABLE 4:** Biochemical parameters of female SD rats treated with sodium p-hydroxybenzoate for 90 days.

Parameter	sodium p-hydroxybenzoate (mg/kg)			
	0	125	250	500
NO. of animals examined	10	10	10	10
Females				
ALT (U/L)	28.1 ± 1.37	27.5 ± 1.51	26.1 ± 2.69	25.9 ± 2.02
AST (U/L)	87.6 ± 7.66	94.9 ± 10.97	87.7 ± 8.26	90.0 ± 12.01
Glucose (mmol/L)	6.42 ± 1.09	6.52 ± 0.62	6.64 ± 0.71	6.58 ± 0.35
Urea (mmol/L)	7.47 ± 2.04	7.59 ± 2.19	7.43 ± 1.22	7.51 ± 0.61
Creatinine (μmol/L)	48.6 ± 12.75	49.9 ± 2.56	49.56 ± 8.65	49.67 ± 5.52
Total cholesterol (mmol/L)	1.58 ± 0.24	1.71 ± 0.15	1.61 ± 0.24	1.74 ± 0.15
Triglyceride (mmol/L)	1.03 ± 0.11	0.99 ± 0.11	1.03 ± 0.21	1.11 ± 0.10
Total Bilirubin (μmol/L)	1.81 ± 0.13	1.7 ± 0.19	1.64 ± 0.17	1.57 ± 0.13*
Total Protein (g/L)	66.6 ± 1.26	67.7 ± 5.25	67.8 ± 3.22	66.8 ± 1.75
Albumin (g/L)	46.6 ± 1.58	46.9 ± 5.30	46.2 ± 3.05	45.5 ± 1.43
Globulins (g/L)	20.2 ± 1.03	20.8 ± 1.23	21.2 ± 1.32	21.0 ± 1.83
A/G (%)	2.32 ± 0.20	2.26 ± 0.30	2.19 ± 0.18	2.17 ± 0.20
Na (mmol/L)	145.1 ± 1.94	145.9 ± 1.86	145.3 ± 1.61	145.4 ± 1.29
K (mmol/L)	4.12 ± 0.82	3.9 ± 0.77	3.96 ± 0.95	4.28 ± 0.70
Cl (mmol/L)	101.3 ± 7.30	97.8 ± 5.79	99.2 ± 7.54	98.3 ± 5.70

AST, aspartate aminotransferase; ALT, alanine aminotransferase; A/G, albumin/globulin ratio; K, potassium; Na, sodium; Cl, chloride.

Data are expressed as means ± SD. *p < 0.05, (significantly different from the control group).

One-way ANOVA test and Bonferroni test.

Thyroid hormone levels, including triiodothyronine (T3), thyroxine (T4) and thyroid-stimulating hormone (TSH), are presented in Table 5. In this experiment, there were no differences between the sodium p-hydroxybenzoate-administered groups and the control group.

**TABLE 5:** Serum thyroid hormone levels of SD rats treated with sodium p-hydroxybenzoate for 90 days.

	sodium p-hydroxybenzoate (mg/kg)			
	0	125	250	500
NO. of animals examined	10	10	10	10
Males				
T3 (ng/ml)	1.14 ± 0.26	1.08 ± 0.23	1.11 ± 0.15	1.10 ± 0.17
T4 (ng/ml)	41.47 ± 3.81	40.78 ± 2.56	42.16 ± 2.82	41.88 ± 2.19
TSH (ng/ml)	4.12 ± 0.24	4.20 ± 0.19	4.16 ± 0.20	4.08 ± 0.24
Females				
T3 (ng/ml)	1.25 ± 0.27	1.27 ± 0.15	1.17 ± 0.13	1.24 ± 0.18
T4 (ng/ml)	28.66 ± 3.66	29.59 ± 3.18	27.64 ± 2.26	28.45 ± 2.88
TSH (ng/ml)	2.37 ± 0.19	2.29 ± 0.12	2.20 ± 0.12	2.19 ± 0.14

T3, triiodothyronine; T4, thyroxine; TSH, thyroid-stimulating hormone.

Data are expressed as means ± SD. *p < 0.05, (significantly different from the control group).

One-way ANOVA test and Bonferroni test.

### Urinalysis

The urinalysis parameters of male and female SD rats are presented in Table 6; Table 7, respectively. Urine density and urine pH did not exhibit significant changes between the control and sodium p-hydroxybenzoate-treated groups. Meanwhile, there were no significant changes in various urinalysis parameters (protein, ketones, glucose, occult blood, urobilinogen, bilirubin) or other urine sediment parameters, such as red blood cells, white blood cells and casts, of the male and female rats treated with sodium p-hydroxybenzoate compared to the control groups.

**TABLE 6:** Urinalysis values of male and female SD rats treated with sodium p-hydroxybenzoate for 90 days.

	sodium p-hydroxybenzoate (mg/kg)			
	0	125	250	500
NO. of animals examined	10	10	10	10
Males				
Urine density	1.016 ± 0.007	1.019 ± 0.007	1.016 ± 0.006	1.015 ± 0.005
Urine pH	7.15 ± 0.53	6.85 ± 0.75	7.2 ± 0.59	7.05 ± 0.55
Females				
Urine density	1.016 ± 0.007	1.018 ± 0.007	1.014 ± 0.004	1.013 ± 0.004
Urine pH	7.10 ± 0.52	6.75 ± 0.63	6.95 ± 0.55	7.00 ± 0.53

Data are expressed as means ± SD. *p < 0.05, (significantly different from the control group).

One-way ANOVA test and Bonferroni test.

**TABLE 7:** Urinalysis values of male and female SD rats treated with sodium p-hydroxybenzoate for 90 days.

Urinalysis parameters	severity scale	sodium p-hydroxybenzoate (mg/kg)							
		Male				Female			
		0	125	250	500	0	125	250	500
Protein									
	−	7/10	6/10	6/10	5/10	6/10	7/10	6/10	6/10
	1+	2/10	2/10	2/10	3/10	2/10	2/10	2/10	2/10
	2+	1/10	2/10	2/10	2/10	2/10	1/10	2/10	2/10
	3+	0/10	0/10	0/10	0/10	0/10	0/10	0/10	0/10
Ketones									
	−	10/10	10/10	10/10	10/10	10/10	10/10	10/10	10/10
Glucose									
	−	10/10	10/10	10/10	10/10	10/10	10/10	10/10	10/10
Occult blood									
	−	10/10	10/10	10/10	10/10	10/10	10/10	10/10	10/10
Urobilinogen									
	+/−	10/10	10/10	10/10	10/10	10/10	10/10	10/10	10/10
Bilirubin									
	−	8/10	8/10	8/10	8/10	9/10	9/10	8/10	8/10
	1+	2/10	2/10	2/10	2/10	1/10	1/10	2/10	2/10
Urinary sediments									
Red blood cell	−	10/10	10/10	10/10	10/10	10/10	10/10	10/10	10/10
White blood cell	−	10/10	10/10	10/10	10/10	10/10	10/10	10/10	10/10
Cast	−	10/10	10/10	10/10	10/10	10/10	10/10	10/10	10/10

No significant differences were noted in any treated groups from control group.

Protein) −: 0 g/L, 1+: 0.3 g/L, 2+: 1.0 g/L, 3+: ≥3.0 g/L.

Ketones) −: 0 mmol/L.

Glucose) −: 0 mmol/L.

Occult blood) −: 0 cells.

Bilirubin) −: 0 μmol/L, 1+: 8.6 μmol/L.

### Absolute and Relative Organ Weights

The absolute and relative organ weights of male and female SD rats, including brain, heart, spleen, lung, liver, kidneys, adrenal, thymus, testis (male), epididymis (male), ovaries (female) and uterus (female), are shown in Tables 8, 9. Changes observed in organ weight are also considered toxicity indicators of compounds administered (Michael et al., 2007; Li et al., 2021), and relative organ weight values have been shown to reflect pathological changes in impaired tissues (Balogun et al., 2014; Li et al., 2014). In males, absolute epididymis weight exhibited a statistically significant (*p* < 0.05) increase in the 500 mg/kg BW/day group compared to the control group. However, since the relative weight of the epididymis did not change and no microscopic changes were observed, this was considered to be due to individual differences and not obviously toxicity-related. In females, a significant increase in the absolute weight of the liver was detected in the 500 mg/kg BW/day group compared with the control, while the relative liver weight did not change. Changes in liver weight can occur in various hepatic diseases, such as steatosis, fibrosis and cirrhosis (Simon et al., 2020), and steatosis can increase liver weight (Molina, 2011). The above results showed that the 500 mg/kg BW/day dose might cause slight liver toxicity in female rats.

**TABLE 8:** Absolute and relative organ weights of male SD rats treated with sodium p-hydroxybenzoate for 90 days.

Males								
Organs Description	sodium p-hydroxybenzoate (mg/kg)				sodium p-hydroxybenzoate (mg/kg)			
	0	125	250	500	0	125	250	500
	Absolute (weight)				Relative (organ weight to body weight)			
Body weight (g)	569.8 ± 50.49	562.6 ± 30.43	570.4 ± 38.99	586.9 ± 15.96				
Brain (g)	2.05 ± 0.35	2.02 ± 0.34	1.97 ± 0.35	1.98 ± 0.28	0.361 ± 0.065	0.359 ± 0.058	0.347 ± 0.062	0.338 ± 0.047
Heart (g)	1.50 ± 0.25	1.59 ± 0.12	1.46 ± 0.06	1.44 ± 0.02	0.262 ± 0.032	0.282 ± 0.018	0.256 ± 0.018	0.245 ± 0.008
Spleen (g)	0.87 ± 0.24	0.87 ± 0.07	0.82 ± 0.06	0.83 ± 0.07	0.153 ± 0.041	0.156 ± 0.014	0.145 ± 0.015	0.142 ± 0.013
Lung (g)	1.88 ± 0.14	1.78 ± 0.10	1.88 ± 0.25	1.83 ± 0.08	0.332 ± 0.037	0.318 ± 0.025	0.330 ± 0.043	0.313 ± 0.018
Liver (g)	12.87 ± 2.31	12.16 ± 0.83	12.78 ± 0.31	13.29 ± 0.66	2.26 ± 0.39	2.17 ± 0.20	2.25 ± 0.15	2.27 ± 0.13
Kidney (g)	3.09 ± 0.28	3.06 ± 0.13	2.92 ± 0.25	3.05 ± 0.07	0.545 ± 0.057	0.546 ± 0.036	0.515 ± 0.059	0.520 ± 0.013
Adrenal (g)	0.072 ± 0.010	0.069 ± 0.014	0.065 ± 0.007	0.065 ± 0.009	0.013 ± 0.002	0.012 ± 0.002	0.011 ± 0.001	0.011 ± 0.001
Thymus (g)	0.447 ± 0.081	0.426 ± 0.052	0.406 ± 0.087	0.480 ± 0.153	0.079 ± 0.016	0.076 ± 0.010	0.072 ± 0.017	0.082 ± 0.026
Testis (g)	3.48 ± 0.24	3.51 ± 0.17	3.52 ± 0.22	3.62 ± 0.17	0.615 ± 0.056	0.626 ± 0.055	0.619 ± 0.050	0.617 ± 0.037
Epididymis (g)	1.28 ± 0.33	1.39 ± 0.18	1.33 ± 0.08	1.57 ± 0.24*	0.224 ± 0.054	0.248 ± 0.035	0.234 ± 0.025	0.268 ± 0.046

Values in the table indicate group means ± SD.; Number of animals (n) = 10.

*p ≤ 0.05 (significantly different from the control group).

**TABLE 9:** Absolute and relative organ weights of female SD rats treated with sodium p-hydroxybenzoate for 90 days

Females								
Organs Description	sodium p-hydroxybenzoate (mg/kg)				sodium p-hydroxybenzoate (mg/kg)			
	0	125	250	500	0	125	250	500
	Absolute (weight)				Relative (organ weight to body weight)			
Body weight (g)	320.2 ± 13.05	309.9 ± 20.27	323.5 ± 37.83	319.5 ± 22.78				
;Brain (g)	1.91 ± 0.29	1.89 ± 0.27	1.95 ± 0.34	1.90 ± 0.35	0.597 ± 0.090	0.608 ± 0.070	0.612 ± 0.142	0.600 ± 0.139
;Heart (g)	1.07 ± 0.09	1.13 ± 0.17	1.03 ± 0.02	1.12 ± 0.17	0.334 ± 0.037	0.364 ± 0.047	0.323 ± 0.035	0.349 ± 0.042
;Spleen (g)	0.562 ± 0.033	0.580 ± 0.109	0.53 ± 0.02	0.61 ± 0.06	0.176 ± 0.008	0.188 ± 0.039	0.165 ± 0.020	0.192 ± 0.021
;Lung (g)	1.26 ± 0.19	1.24 ± 0.26	1.16 ± 0.06	1.26 ± 0.15	0.394 ± 0.066	0.405 ± 0.099	0.362 ± 0.044	0.398 ± 0.066
;Liver (g)	7.64 ± 0.55	8.01 ± 0.70	8.08 ± 0.36	8.38 ± 0.48*	2.39 ± 0.19	2.72 ± 0.25	2.53 ± 0.34	2.52 ± 0.28
;Kidney (g)	1.98 ± 0.33	1.95 ± 0.23	2.05 ± 0.33	2.02 ± 0.32	0.621 ± 0.109	0.633 ± 0.096	0.646 ± 0.148	0.636 ± 0.116
;Adrenal (g)	0.075 ± 0.017	0.069 ± 0.013	0.067 ± 0.007	0.084 ± 0.009	0.024 ± 0.006	0.022 ± 0.004	0.021 ± 0.003	0.027 ± 0.003
;Thymus (g)	0.379 ± 0.063	0.388 ± 0.088	0.424 ± 0.068	0.368 ± 0.114	0.118 ± 0.020	0.126 ± 0.033	0.133 ± 0.026	0.115 ± 0.032
;Ovary (g)	0.134 ± 0.010	0.126 ± 0.017	0.119 ± 0.015	0.132 ± 0.011	0.042 ± 0.004	0.041 ± 0.005	0.038 ± 0.006	0.042 ± 0.004
;Uterus (g)	0.790 ± 0.311	0.826 ± 0.234	0.884 ± 0.160	0.642 ± 0.107	0.246 ± 0.093	0.267 ± 0.079	0.276 ± 0.055	0.202 ± 0.035

Values in the table indicate group means ± SD.; Number of animals (n) = 10.

*p ≤ 0.05 (significantly different from the control group).

### Histopathological Survey and Gross Pathological Analysis

**FIGURE 4:**
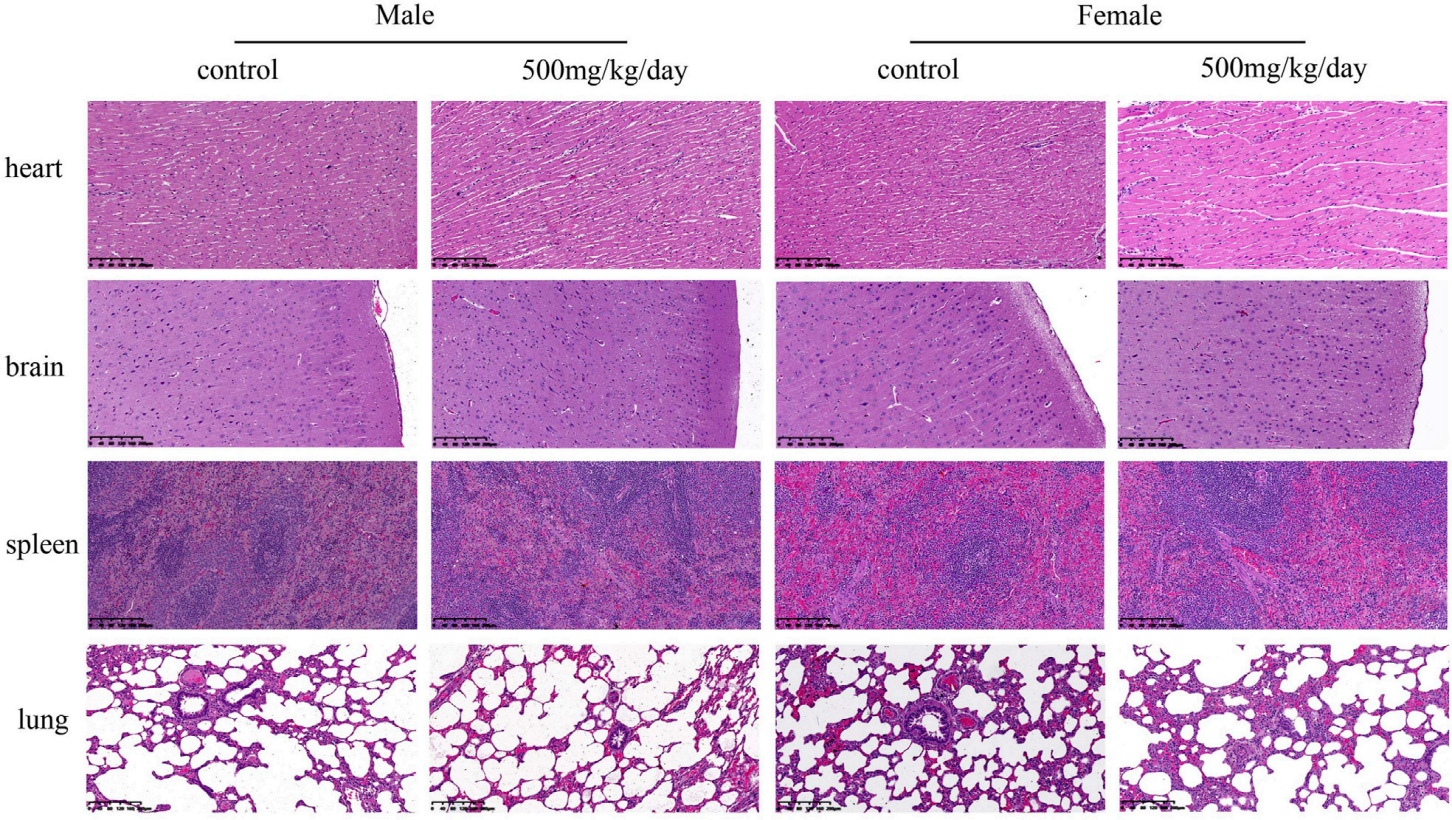
Representative microscopic evaluation (H&E staining) of heart, brain, spleen and lung tissues of rats administered control and 500 mg/kg BW/day sodium p-hydroxybenzoate to male and female SD rats (scale bar = 200 μm).

**FIGURE 5:**
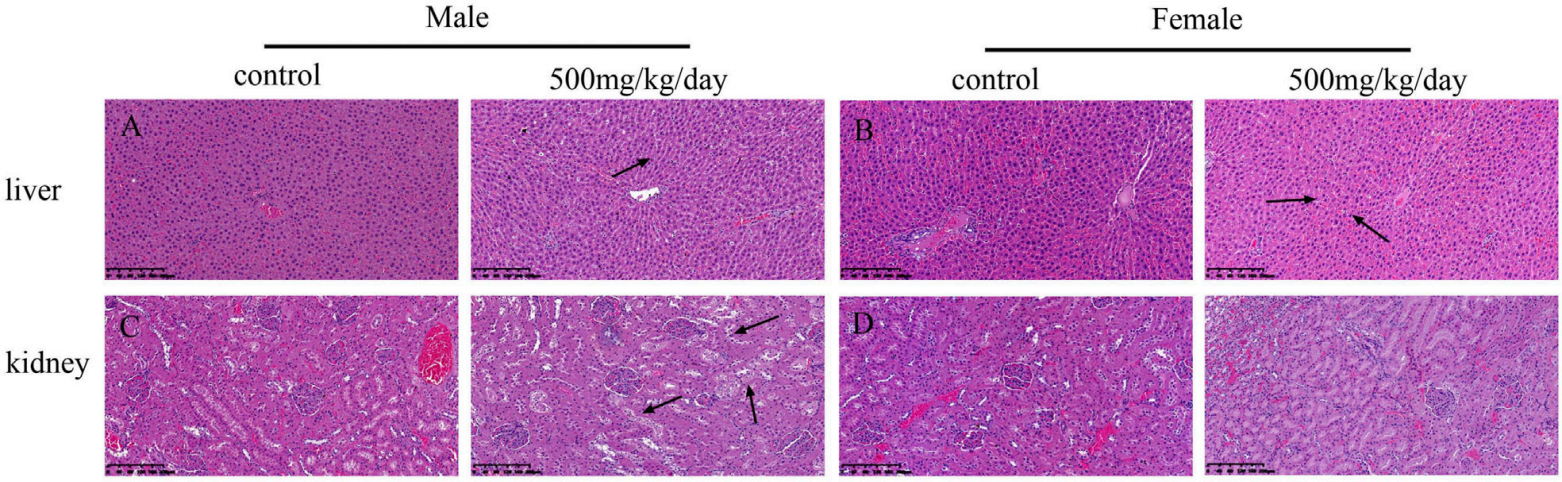
Microscopic evaluation (H&E staining) of liver and kidney tissues from rats administered control and 500 mg/kg BW/day sodium p-hydroxybenzoate to male and female SD rats (scale bar = 200 μm). Panel **(A)** shows normal liver architecture in control group of males, at 500 mg/kg, vacuolated degeneration was seen (arrow heads). Panel **(B)** shows normal liver architecture in control group of males, at 500 mg/kg, vacuolated degeneration was seen (arrow heads). Panel **(C)** shows normal kidney architecture in control group of males, at 500 mg/kg slight edema and degeneration of renal tubular epithelial cells was seen (arrow heads). Panel **(D)** shows no obvious changes were observed in the kidney in females compared to that in the control group.

The necropsy results indicated no treatment-related changes had occurred in any sodium p-hydroxybenzoate-treated group. The histopathological findings are shown in Table 10. In the heart, minimal edema of the myocardial interstitium was observed in some male rats. Pulmonary and splenic congestion was observed in both sexes in the control and treatment groups. Local minimal inflammation was found in the 500 mg/kg BW/day group in both males and females in the stomach. These histopathologies were minimal and showed no significant treatment relationship between the control group and treated group. Tissue images from the control and high-dosing groups stained with H&E are shown for major representative organs (heart, brain, spleen and lung) (Figure 4). Slight edema and degeneration of renal tubular epithelial cells were observed in the 500 mg/kg BW/day male group. In the liver, hepatic sinusoid congestion was observed in both sexes in the control and treatment groups. Vacuolated degeneration was found in the 500 mg/kg BW/day group in both males and females. Tissue images of liver and kidney from the control and high-dosing groups stained with H&E are shown in Figure 5.

**TABLE 10:** Histopathological findings of SD rats treated with sodium p-hydroxybenzoate for 90 days.

	sodium p-hydroxybenzoate (mg/kg)					
Organs	Findings	NO. of animals examined	0	125	250	500
			10	10	10	10
Males						
Heart	Edema of myocardial interstitium, minimal		0	−	−	2
Liver	Congestion, hepatic sinusoids, slight/		1	1	1	2
	Vacuolar degeneration, minimal		0	0	0	2
Lung	Congestion, minimal		2	2	2	3
spleen	Congestion, minimal		1	2	2	2
stomach	Inflammation, minimal		0	−	−	2
intestine	Infiltration, lymphocytes cell, minimal		2	2	2	3
Females						
liver	Congestion, hepatic sinusoids, slight/		0	−	−	1
	Vacuolar degeneration, minimal		0	0	0	3
lung	Congestion, moderate		2	2	1	3
spleen	Congestion, minimal		2	3	3	3
stomach	Erosion/Ulcer, minimal		0	−	−	2
intestine	Infiltration, lymphocytes cell, minimal		3	3	3	4

## Discussion

Sodium p-hydroxybenzoate is a sodium salt of p-hydroxybenzoic acid (p-HBA). In our previous acute oral toxicity experiment, the LD50 of p-hydroxybenzoic acid was greater than 2,000 mg/kg, while the LD50 of sodium p-hydroxybenzoate was greater than 5,000 mg/kg, the safety of oral administration is improved. However, information regarding subchronic toxicity is unclear. Therefore, the aim of this study was to conduct a comprehensive toxicological evaluation of sodium p-hydroxybenzoate to determine its safety for humans at chronic doses. In the present study, the 90-days repeated-dose subchronic oral toxicity of sodium p-hydroxybenzoate in SD rats was determined to evaluate its safety profile according to the OECD guideline 408. Subchronic oral toxicity studies are usually based on LD50 and ED50 calculated from acute toxicity tests (Ntchapda et al., 2014). The highest dose is usually 5–15% of LD50 (Wei et al., 2019), and the lowest dose is usually higher than the daily possible intake. As a result, in the subchronic toxicity study of sodium p-hydroxybenzoate, 0, 125, 250, and 500 mg/kg BW/day doses were used. At the end of the treatment regimen, all animals survived until necropsy without any clinical changes. Several studies have demonstrated that adverse effects of p-HBA cause male infertility and female breast cancer (Asif et al., 2021). These results indicated that the safety of sodium p-hydroxybenzoate is higher than that of p-HBA.

Body weight and food intake are typical indicators of adverse effects of drugs on the body (Bilan et al., 2011). Changes in body weight imply that the administered chemical might be toxic to the experimental animals (Teo et al., 2002). In the present experiment, our results showed that no significant differences in body weight or food consumption developed between the control and treated groups during the experimental period. These findings suggested that sodium p-hydroxybenzoate does not influence the body weight or food consumption of experimental animals in the present study. However, the final weight difference is big among the male and female rats. Despite this, the weight gain of males and females rats has sustained the normal range during the study period (Lira et al., 2020; Won et al., 2021). The basal metabolism and energy expenditure of male rats is higher than that of female rats, which may be the reason for the large difference in final body weight. In addition, all experimental subjects exhibited a similar and slightly decreasing trend in feed consumption, we speculate that except for satisfying own consumption and basal metabolism, some feed was stored as fat and other substances in the body. The foraging amount decreased with the storage amount over time.

Hematological parameters are important indicators of the physiological and pathological status of the body, such as white blood cell (WBC), red blood cell count (RBC), and platelet count (PLT) (Li et al., 2021). Significant changes (either increases or decreases) are due to the influence of the administered compound on the animals (Obakiro et al., 2021). In the present study, there were several statistically significant differences detected in hematological parameters at the end of the experiment in males in the 500 mg/kg BW/day dose group compared to controls, such as white blood cells (WBCs) and prothrombin time (PT). It is well documented that white blood cells are effective markers of inflammation (Karino et al., 2015). These results indicated that the 500 mg/kg BW/day dose may cause hematological toxicity in male rats.

Clinically, various adverse effects of nonsteroidal anti-inflammatory drugs have been reported, including gastrointestinal symptoms, renal injury, liver injury and other symptoms. Therefore, evaluating liver function is very important and is usually assessed by ALT and AST levels (Olorunnisola et al., 2012). Meanwhile, hematological and biochemical parameters also reflect potential lesions of the liver and kidney (Gao et al., 2017; Kapoor et al., 2021). ALT (Costa-Silva et al., 2008) is a sensitive indicator of liver injury and hypertrophy, while AST is an important indicator of liver, kidney, muscle and heart diseases (Adeyemi et al., 2010; Xiang et al., 2015; Garcia et al., 2016; Yi et al., 2018). Regarding the biochemical parameters, no statistically significant differences were observed in the levels of ALT and AST, but slight histopathological changes were observed at the 500 mg/kg BW/day dose in the liver, indicating that the highest dose of sodium p-hydroxybenzoate may cause side effects on the liver. The kidney is also a vital target organ of toxic compounds because toxins usually accumulate in its tubules (Akindele et al., 2014). There are many biomarkers of renal damage, such as urea, creatinine and uric acid (Gowda et al., 2010). In the present study, urea was significantly reduced in the 250 mg/kg BW/day group in males compared to the control group, which is considered incidental, as no differences were noted in the female group and the change was not dose-related. Some histopathological changes were observed, such as slight edema and degeneration of renal tubular epithelial cells in the 500 mg/kg BW/day group in males. These changes were considered adverse effects of sodium p-hydroxybenzoate in the kidneys of males.

Usually, comparison of the organ weights was used to evaluate the toxic effect of the test compound in toxicological experiments (Michael et al., 2007). In the present study, the absolute weight of the liver in females was significantly increased in the 500 mg/kg BW/day group compared to the control group. The changes observed in absolute weight of the liver might be toxicologically relevant.

In the liver, vacuolated degeneration was observed in both sexes in the 500 mg/kg BW/day group. Meanwhile, the number of white blood cells (WBCs) was increased in males, and the absolute weight of the liver was increased in females. All of these changes were likely related to sodium p-hydroxybenzoate administration.

## Conclusion

In conclusion, sodium p-hydroxybenzoate administration resulted in slight hepatic or kidney toxicities in both males and females receiving 500 mg/kg BW/day. These changes were systemically observed and considered to be adverse effects associated with sodium p-hydroxybenzoate. Therefore, in the 90-days subchronic toxicological study, the NOAEL for sodium p-hydroxybenzoate was determined to be 250 mg/kg BW/day for both male and female SD rats. The present study is the first report of a safety assessment of sodium p-hydroxybenzoate. This paper found that sodium p-hydroxybenzoate is safe and has little effect on vital organs, so we speculate that sodium p-hydroxybenzoate is higher security than p-HBA. The clinical use of sodium p-hydroxybenzoate requires a good level of safety, and these results provide a foundation for future preclinical and clinical studies. We will investigate the carcinogenic studies in the future, enabling this research to become more complete.

## Data Availability

The original contributions presented in the study are included in the article/supplementary material, further inquiries can be directed to the corresponding authors.

## Abbreviations

A/G, albumin/globulin ratio; ALT, alanine aminotransferase; APTT, activated partial thromboplastin time; AST, aspartate aminotransferase; BAS, basophil count; Cl, chloride; EOS, eosinophil count; HCT, hematocrit; HGB, hemoglobin; K, potassium; LYM, lymphocyte count; MCH, mean corpuscular hemoglobin; MCHC, mean corpuscular hemoglobin concentration; MCV, mean corpuscular volume; MON, monocyte count; Na, sodium; NEU, neutrophil count; NOAEL, the no-observed-adverse-effect level; PLT, platelet count; PT, prothrombin time; RBC, red blood cell count; RDW%, red blood cell distribution width; WBC, white blood cell count.
